# Harnessing the early post-injury inflammatory responses for cardiac regeneration

**DOI:** 10.1186/s12929-017-0315-2

**Published:** 2017-01-13

**Authors:** Bill Cheng, H. C. Chen, I. W. Chou, Tony W. H. Tang, Patrick C. H. Hsieh

**Affiliations:** 1 Institute of Biomedical Sciences, Academia Sinica, 128 Academia Road, Sec. 2, Nankang District Taipei, 115 Taiwan; 2 Graduate Institute of Life Sciences, National Defence Medical Center, Taipei, 114 Taiwan; 3 Program in Molecular Medicine, National Yang Ming University, Taipei, 112 Taiwan; 4 Graduate Institute of Medical Genomics and Proteomics, and Institute of Clinical Medicine, College of Medicine, National Taiwan University, Taipei, 100 Taiwan; 5 Department of Surgery, National Taiwan University Hospital, Taipei, 100 Taiwan

**Keywords:** Heart regeneration, Inflammation, Macrophage

## Abstract

Cardiac inflammation is considered by many as the main driving force in prolonging the pathological condition in the heart after myocardial infarction. Immediately after cardiac ischemic injury, neutrophils are the first innate immune cells recruited to the ischemic myocardium within the first 24 h. Once they have infiltrated the injured myocardium, neutrophils would then secret proteases that promote cardiac remodeling and chemokines that enhance the recruitment of monocytes from the spleen, in which the recruitment peaks at 72 h after myocardial infarction. Monocytes would transdifferentiate into macrophages after transmigrating into the infarct area. Both neutrophils and monocytes-derived macrophages are known to release proteases and cytokines that are detrimental to the surviving cardiomyocytes. Paradoxically, these inflammatory cells also play critical roles in repairing the injured myocardium. Depletion of either neutrophils or monocytes do not improve overall cardiac function after myocardial infarction. Instead, the left ventricular function is further impaired and cardiac fibrosis persists. Moreover, the inflammatory microenvironment created by the infiltrated neutrophils and monocytes-derived macrophages is essential for the recruitment of cardiac progenitor cells. Recent studies also suggest that treatment with anti-inflammatory drugs may cause cardiac dysfunction after injury. Indeed, clinical studies have shown that traditional ant-inflammatory strategies are ineffective to improve cardiac function after infarction. Thus, the focus should be on how to harness these inflammatory events to either improve the efficacy of the delivered drugs or to favor the recruitment of cardiac progenitor cells.

## Background

Myocardial infarction (MI) continues to be a major cause of morbidity and mortality in many countries. In the United States, MI is responsible for more deaths than cancer and traffic accidents combined [1]. Although significant advances have been made in identifying potential drug targets, there is still no specific treatment that targets myocardial injury in patients with MI [2, 3]. An enormous body of evidence indicates that the inflammatory responses that occur after MI play critical roles in the overall cardiac output of the infarcted heart. Thus, recent efforts by the scientific community and industry have focused on understanding how the inflammatory activities exerted by the recruited immune cells influence the microenvironment of the infarcted heart in order to achieve the desired clinical outcome.

Clinically, MI can be characterized into two main phases, cardiac ischemia and reperfusion [4]. In cardiac ischemia, patients usually first experience onset of chest pain at the moment that an occlusion has happened in one of the coronary arteries. Subsequently, upon arrival in hospital, patients receive thrombolytic therapy or percutaneous coronary intervention to allow cardiac reperfusion to happen. Even after oxygenation is restored during reperfusion, cardiomyocytes still experience cell apoptosis due to profound inflammation. Since the adult mammalian heart has very little regenerative capacity, the healing process of the infarcted myocardium is dependent on the immune cells that are recruited to the infarcted heart, which eventually lead to the formation of a collagen-based scar. The main role of the scar is to replace the dead cardiomyocytes thereby preserving the structural integrity of the left ventricles. However, recent studies have shown that the recruited immune cells, particularly monocytes and their derivative, macrophages, release cytokines and proteases that induce apoptosis in healthy cardiomyocytes. Thus, as more cardiomyocytes undergo cell apoptosis, the size of scar tissue increases, which is the cause of cardiac fibrosis that is characterized by loss of cardiac muscle elasticity and eventually heart failure.

Previously, anti-inflammatory therapeutics that target the recruited monocytes have been considered as a suitable therapy to prevent further weakening of the myocardium after MI. In recent clinical trials, however, many of the anti-inflammatory drugs such as Darapladib failed to reach primary end-point [5]. In addition, small molecules like metformin were shown to induce undesired side-effects in patients [6]. Apart from poor drug retention in the heart, it is becoming clear that the immune cells also have reparative roles in heart healing. Recent studies on lower vertebra, zebra fish and the neonatal heart of mouse, have revealed that inflammation, particularly caused by macrophages, is an essential component of tissue regeneration [7, 8]. Depletion of monocytes in neonatal mice before heart injury abolishes subsequent organ regeneration, resulting in excessive scarring and compromised cardiac function typical of an adult response [9]. Therefore, cardiac inflammation has more complex roles than previously thought post-MI. In this review, we focus on the roles of key immune cells that participate in the early stage of healing after MI, as well as novel strategies that utilize existing inflammatory responses with an eye to achieving desired clinical outcomes in patients with MI.

### Neutrophils

Immediately after cardiac ischemic injury, neutrophils are the first innate immune cells recruited to the ischemic myocardium within the first 24 h post-MI, especially after reoxygenation is achieved. From a classic immunological perspective, neutrophils are known to play critical roles in preventing bacterial infection during the wound healing process. Patients that have low neutrophil counts or lack functional neutrophils often suffer from severe bacterial and fungal infections after a non-sterile injury has taken place [10]. Physiologically, neutrophils are programmed to undergo apoptosis after infiltrating into the injured myocardium, in which the apoptotic neutrophils attract macrophage recruitment and promote the clearance of apoptotic cells in the injured tissue [11]. Therefore, in principle, these apoptotic neutrophils are negative regulators of cardiac inflammation as macrophages may remove cell debris by releasing anti-inflammatory factors such as IL-10 [12]. In clinical situations, however, the lifespan of neutrophils at the infarct area is prolonged due to the effect of tumor necrosis factor (TNF)-α and interleukin (IL)-1β [13]. These ‘surviving’ neutrophils then secrete proteases such as complement component C5a that promote cardiac remodeling and chemokines that further potentiate leukocyte recruitments. Moreover, the infiltrated neutrophils can also induce apoptosis in healthy cardiomyocytes through the release of reactive oxygen species (ROS).

Initially, neutrophils are guided to the injured myocardium by the gradient of the released mitochondrial damage-associated molecular patterns (DAMPs). Upon the ischemic and reperfusion injury, ruptured cells release all their cellular contents, including mitochondria into the circulation. Since mitochondria and mitochondrial DNA are structurally similar to their bacterial counterparts, their presence in circulation is immediately detected by neutrophils. Two of the neutrophil membrane receptors, formyl peptide receptor 1 (FR1) and Toll-like receptor 9 (TLR9) can recognize the presence of the formylated peptide component of the mitochondrial membrane and mitochondrial DNA, respectively [14]. The binding of the released mitochondrial components to neutrophil receptors triggers neutrophil activation and promotes cell extravasation into the injured myocardium. Additionally, the mitochondrial DAMPs and other released cellular components create a signaling gradient, allowing the nearby neutrophils to precisely home to the targeted site [15]. DAMPs released by ruptured cardiomyocytes also induce cardiac mast cell degranulation, resulting in the release of contents such as histamine, TNF-α, and IL-1β. These factors activate cardiac endothelial cells, and induce the upregulation of membrane surface receptors that facilitate neutrophil extravasation through the endothelium to reach the targeted site.

Although neutrophils seem to have no direct role in cardioprotection, lack of neutrophils results in worse cardiac function and increased fibrosis and their depletion does not accelerate heart healing after MI [16]. It is well-established that post-MI inflammation resolution is characterized by the local conversion of pro-inflammatory M1 macrophages into reparative M2 macrophages. Traditionally, it has been thought that the M1/M2 stereotype macrophages are influenced by the different ratio of cytokines present in the myocardial microenvironment [17]. A more recent study, however, demonstrated that neutrophils have a direct influence on the polarization of macrophages after MI [18]. In neutrophil-deficient mice with MI, it was noticed that there was significantly fewer splenic Ly6C^high^ monocytes in the heart compared to the wild-type mice with MI. Although there were more reparative M2 macrophages in the heart of neutrophil-deficient mice compared to wild-type mice, these macrophages had reduced expression of phagocytosis receptor myeloid-epithelial-reproductive tyrosine kinase (MertK) [18]. MertK is a marker of reparative M2 macrophages, which mediate clearance of apoptotic cells. Thus, the low expression of MertK in neutrophil-deficient mice results in insufficient clearance of apoptotic cells by the reparative M2 macrophages, which leads to delayed inflammation resolution after MI. Interestingly, circadian oscillations of neutrophil recruitment to the heart also determine infarct size, healing, and cardiac function after MI [19]. The study revealed that MI that happens during sleep-to-wake transition leads to excessive cardiac neutrophil recruitment, larger infarct size, and worsened heart function.

### Monocytes and macrophages

Traditionally, it was thought that monocyte recruitment happened immediately after neutrophils had infiltrated the injured myocardium. However, intravital microscopy of the beating mouse heart has shown that monocytes are detected within 30 min after MI [20]. Unlike neutrophils, however, the number of monocytes being recruited to the heart does not peak within the first 24 h post-MI. Instead, immunohistological staining of heart tissue sections of deceased patients revealed the maximum number of monocytes being recruited to the heart happens at 72 h post-MI [21]. Moreover, the extravasation of monocytes begins at the remote area, where healthy myocardial tissue is present. Subsequently, the monocytes migrate through the border zone and accumulate at the infarct area. Such a migration pattern explains why the targeting resolution of inflammation is a viable therapeutic strategy in heart healing, since monocytes are known to secret inflammatory cytokines and proteases that are detrimental to cardiomyocytes [22]. Thus, if inflammation is prolonged, which is commonly seen among patients with MI, the secreted factors will not only further damage the surviving cardiomyocytes in the infarct area, they will also harm the healthy cardiomyocytes at the remote and border zones.

In the context of MI, monocytes recruited to the heart in patients with MI can be divided into two subpopulations, Ly6C^high^ and Ly6C^low^. The Ly6C^high^ monocytes are commonly known as pro-inflammatory monocytes, whereas the Ly6C^low^ monocytes are sometimes known as resident monocytes because of their capacity to accumulate regardless of inflammation [23]. Currently, it is not clear which monocyte subset infiltrates the heart immediately post-MI. However, it is well-established that chemokine monocyte chemotactic protein (MCP)-1 drives the recruitment of Ly6C^high^ monocytes to the heart within the first 24 h post-MI [24]. Days later, once the inflammation is starting to resolve, the number of Ly6C^high^ monocytes in the heart or blood decreases, whereas the number of Ly6C^low^ monocytes increases [25]. This conversion corresponds to the presence of proinflammatory M1 macrophages in the heart early after injury, and reparative M2 macrophages at the later stage of heart healing [25]. It is not certain whether M2 macrophages are trans-differentiated directly from M1 macrophages, or whether the trans-differentiation requires the conversion from Ly6C^high^ to Ly6C^low^ monocytes. However, there is strong evidence that both the Ly6C^high^ and Ly6C^low^ monocytes arise from the same progenitor cells [26], and that through a nuclear receptor subfamily 4 group A member 1 (NR4A1)-dependent transcriptional program, Ly6C^high^ monocytes differentiate into Ly6C^low^ monocytes [27].

In humans, cardiac monocytes are also classified into two subsets based on the expression level of CD14 and CD16. The CD14^+^ CD16^−^ and CD14^+^ CD16^+^ monocytes are the human analogues of mouse Ly6C^high^ and Ly6C^low^ monocytes, respectively. Clinical data indicate that at the early stage of MI in human patients, ~85% of the monocytes detected in the heart are CD14^+^ CD16^−^ monocytes which exhibit pro-inflammatory activity [28]. Similar to the time course seen in the murine model of MI, as the inflammation resolves the monocyte population starts to shift towards the CD14^+^ CD16^+^ subset [28]. It was demonstrated that at 5-7 days post-MI, 60% of CD14^+^ CD16^−^ and 40% of CD14^+^ CD16^+^ are accumulated in the infarct area [21].

### Cardiac resident macrophages

Previously, it was thought that the profound inflammation that happens in the heart after MI is heavily influenced by the recruited monocyte-derived macrophages. However, recent studies have demonstrated the existence of cardiac macrophages derived from embryonic precursors that are termed resident macrophages [29]. Unlike the monocyte-derived macrophages, cardiac resident macrophages are established in the heart during embryonic development and are easily detected at E10.5 [29]. Furthermore, these resident macrophages are yolk sac-derived since they are detected in the heart prior to fetal liver hematopoiesis [30]. Similar to other embryonic yolk sac macrophages in other tissues, cardiac resident macrophages have the expression pattern MHC-II^low^, CX3CR1^high^, F4/80^high^, and CD11b^low^. Studies on the healthy heart of CX3CR1^GFP/+^ mice reveal a large number of macrophages are in direct contact with myocytes and endothelial cells [31]. Under non-pathological conditions, cardiac resident macrophages are considered non-inflammatory. The cells have low expression level of Ly6C markers and have a set of 22 upregulated genes (including Mrc1, CD163, and Lyve-1) that are characteristics of activated M2 macrophages [31]. Interestingly, cardiac resident macrophages are also found to express some inflammatory genes, including IL-1β, which highlights the limitations of the M1/M2 classification [32]. The function of cardiac resident macrophages in healthy heart is still under investigation, although it has been speculated that these cells may be involved in preventing bacterial infection, regulating angiogenesis, and matrix protein turnover [32].

The resident macrophages within the heterogeneous population of macrophages in the infarcted heart can be distinguished by the expression level of the surface marker CCR2 [32]. Unlike the monocyte-derived macrophages, cardiac resident macrophages have very low level of expression of CCR2. The chemokine receptor, CCR2, also known as CD192, is a key receptor that facilitates monocyte extravasation through the recognition of MCP-1 [33]. Studies of the proliferation marker Ki-67 revealed that cardiac resident macrophages undergo rapid proliferation to increase their numbers in the heart after MI, whereas no proliferative activity is detected in the recruited monocytes after they differentiate into macrophages [29]. Although monocyte-derived macrophages play important roles in coordinating cardiac inflammation, their roles in antigen sampling and efferocytosis are less critical than the resident macrophages [34]. Mice that lack circulating monocytes are found to have fewer inflammatory activities associated with cardiac pathology after injury [35], suggesting that excessive expansion of macrophage populations can have a detrimental effect on heart healing. Additionally, the loss of Ly6C^high^ monocytes also prevents hypertension-induced cardiac fibrosis and improves left ventricle function after MI [36]. Indeed, it has been found that cardiac resident macrophages are more efficient at removing apoptotic cardiomyocytes, thus promoting the resolution of cardiac inflammation [37]. Like the reparative M2 macrophages, the resident macrophages also have a high expression level of MertK, and the loss of this receptor leads to increased neutrophil persistence and decreased level of IL-10 in the myocardium [37]. Thus, a good anti-inflammatory strategy for treating patients with MI in the future would be to selectively target the recruited monocytes, without affecting the activity of resident macrophages.

### Macrophages and endogenous stem cells

Since adult mammalian hearts have poor regenerative capability, the ultimate goal of any cardioprotective treatment is to achieve a substantial level of cardiac muscle regeneration. Genetic fate-mapping study of adult murine hearts demonstrates that there are stem cells or precursor cells present in the heart that contribute to the replacement of adult mammalian cardiomyocytes after myocardial infarction [38]. As also highlighted above, two populations of macrophages, M1 and M2, participate in creating the phase 1 (day 1-3 after MI) and phase 2 (day 4-7 after MI) inflammatory microenvironments in the infarct area respectively [39–41]. The M1 macrophages that are dominant in phase 1 of MI phagocytose cell debris in the infarct zone and secrete pro-inflammatory cytokines, such as TNFα, IL1β, IL6 and IL10 [41]. In contrast, M2 macrophages, the major macrophages in phase 2 of MI, promote collagen deposition and angiogenesis to the infarct area [40]. The inflammatory microenvironments not only activate cardiofibroblasts for myocardium remodelling, but also activate endogenous stem cells for heart regeneration, either by cell fusion or transdifferentiation [42–44]. Despite that the endogenous cardiac progenitor cells (CPCs) are activated in response to heart damage [42, 43], nevertheless an as yet unclear interaction between CPCs and macrophages in the infarct area remains to be elucidated. One key factor that bridges CPCs and macrophages in the injured heart is prostaglandin E_2_ (PGE_2_), whose release from the injured heart regulates macrophage populations and exerts a salutary effect on the myocardium [45–47].

PGE_2_ in the injured site binds to the G protein-coupled receptor E prostanoid 2 (EP2) on monocytes to suppress the maturation of these monocytes to M1 macrophages through activating the downstream cyclic AMP (cAMP)/protein kinase A (PKA) signalling [48]. PGE_2_ also activates EP2/EP4 receptors, which induce upregulation of cAMP and its downstream cAMP responsive element binding (CREB)/transcriptional coactivators 2 and 3 (CRTC2/3)-mediated induction of Krupple like factor 4 (KLF4) to promote polarization of M2 macrophages [47]. Therefore, strategies modulating the balance of M1/M2 macrophages such as by PGE2 treatment may create a favourable inflammatory microenvironment in the infarct zone to promote heart repair and regeneration after MI [40, 49].

### Harnessing early cardiac inflammation

Despite its poor clinical outcome in recent trials in patients with MI, anti-inflammatory therapeutic strategy is still considered to be a viable option for controlling the size of the infarcted area. From the recent advances in understanding the roles that the innate immunity plays in post-MI, it is increasingly clear that downregulating the inflammatory activity exerted by recruited monocyte-derived macrophages would promote better cardiac output in patients with MI [34]. Since macrophages have different roles at different time points after MI, future therapeutic strategies should focus on minimising the inflammatory effects rather than completely inhibiting the entire inflammatory activities. Thus, an ideal treatment should be able to assist the recruited immune cells to create an inflammatory microenvironment that is favourable for cardiac regeneration but with minimal interferences to their inflammatory activities at a specific time point. Here, we present two therapeutic strategies that harness the inflammatory events that happen after MI to achieve cardioprotection and to improve cardiac regeneration.

### Biomimicking platelet-monocyte interactions

Similar to other cardioprotective drugs, anti-inflammatory therapeutics that are designed for cardioprotection in the heart have poor targeting for the organ itself. Although these drugs have high specificity for their designed targets, they have poor retention in the heart after MI. Consequently, poor targeting has been a key reason that explains why some of these drugs could not be translated into clinical practice [50]. The issue of poor targeted drug delivery for infarcted hearts is evident by a recent clinical trial of cyclosporine in patients with MI [51]. Despite the drugs were encapsulated in PEGylated liposomes, the results from the trial revealed that most of the administered cyclosporine was distributed in other organs rather than in the heart. Poor drug targeting not only cannot improve overall cardiac output, but also can induce undesired side-effect in other organs [52]. Thus, there is an urgent need to develop a novel delivery strategy that can maximize the overall efficacy of the delivered drugs for cardioprotection.

**Fig. 1:**
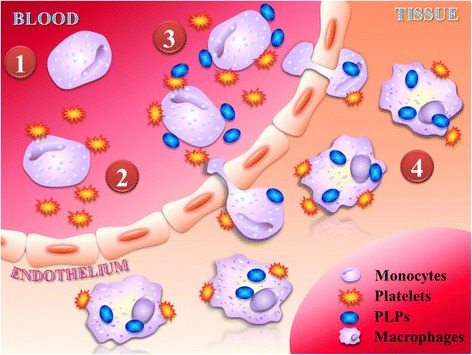
Platelet-like proteoliposomes enhance the targeting specificity for infarcted heart through biomimicking platelet interactions with circulating monocytes. (1) Platelets adhere to the surface of recruited monocytes during the development of MI. (2) Accordingly, platelet-monocyte aggregates will undergo extravasation. (3) It is hypothesized that platelet-like proteoliposomes (PLPs) will interact with monocytes in a similar way to platelets. (4) Once crossing the endothelium, the PLPs are expected to be phagocytized by monocyte-derived macrophages

It has recently been proposed that platelet-like proteoliposomes (PLPs) that can biomimic platelet interactions in circulating monocytes act as a novel way of delivering anti-inflammatory drugs to the infarcted heart [53]. Clinically, platelets are found to interact with surfaces of the recruited monocytes in patients with MI to form platelet-monocyte aggregates, which have been used as an early detection biomarker and for monitoring the progression of the disease [54]. The biological significance of the binding between platelets and monocytes is still not known, but it has been suggested to facilitate monocyte extravasation into tissue [55]. Similar to circulating platelets, PLPs show strong binding affinity for monocytic cell lines, but not for endothelial cells. More importantly, PLPs are able to infiltrate into the infarct area in large number by anchoring on the surfaces of the recruited monocytes. Therefore, in this monocyte-mediated delivery strategy, the host monocytes are used as “shuttle buses” to carry the PLPs and their cargoes directly to the heart (Fig. 1). Such a delivery strategy is more effective than the current delivery strategy which relies on the presence of an enhanced permeability and retention (EPR) effect [53]. A recent study on nanoparticle distribution in the murine model of I/R has revealed that EPR effect starts to diminish after 24 h post-infarction [56], which explains why so many cardioprotective drugs have poor retention in the heart. Therefore, unlike in cancer, EPR effect only exists for a short duration after MI, which is insufficient for meaningful cardioprotection and preventing remodelling, which takes place over days to weeks.

Another advantage of the monocyte-mediated strategy is the selectivity for the recruited monocyte-derived macrophages. Since PLPs could only infiltrate the infarcted heart through interactions with recruited monocytes, the particles themselves are immediately phagocytized by the recruited monocyte-derived macrophages upon entering the myocardium. Consequently, PLPs have less chance to contact with the cardiac resident macrophages after MI, allowing the encapsulated drugs to release within the recruited monocyte-derived macrophages only. This monocyte-mediated strategy has opened up a new paradigm in drug delivery, as it is the first reported case of EPR-independent drug delivery to the heart, and that the delivery vehicle specifically targets the recruited monocytes.

### PGE_2_ and M2 macrophage polarization

**Fig. 2:**
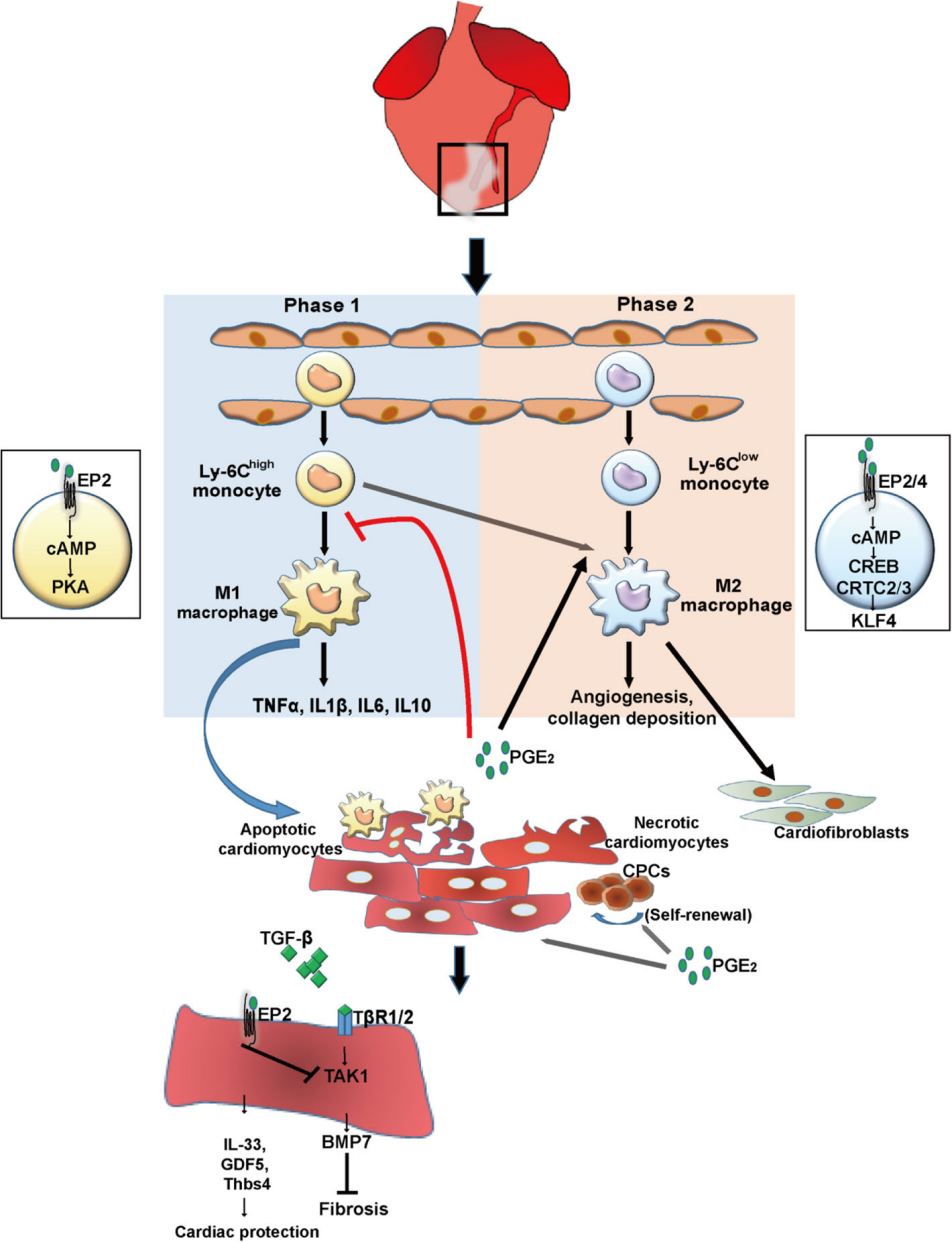
Effects of prostaglandin E_2_ on macrophages, cardiomyocytes and cardiac progenitor cells after myocardial infarction. Ly6C^high^ monocytes undergo maturation to generate M1 macrophages during the phase 1 of inflammation in the infarct area and perform phagocytosis to clean cell debris and produce pro-inflammatory cytokines TNFα, IL1β, IL6 and IL10. Maturation of M1 macrophages is inhibited by PGE_2_ via the EP2/cAMP/PKA pathway. Ly6C^low^ monocytes undergo M2 macrophage polarization during the phase 2 of inflammation which is promoted by PGE_2_ through the EP(2/4)/cAMP/CREB/CRTC(2/3)/KLF4 pathway

Traditionally, PGE_2_ is considered a proinflammatory molecule. However, it has recently been suggested that PGE_2_ may modulate the inflammatory microenvironment for tissue regeneration through regulating macrophage subtypes [57]. Intraperitoneal injection of PGE_2_ in a murine model of MI has been shown to promote replenishment of cardiomyocytes from endogenous CPCs by down-regulation of TGF-β signalling in cardiomyocytes [49]. The effect of PGE2 on CPCs is mediated through interaction with the EP2 receptor [49]. However, the molecular mechanism underpinning the TGF-β-mediated salutary effect of PGE_2_ on CPCs is unclear. One possible mechanism is through inhibition of the TGF-β/TGF-β type 2 receptor (TβR2)/TGF-β-activated kinase 1 (TAK1) signalling in cardiomyocytes, which leads to upregulation of bone morphogenetic protein 7 (BMP7) and thus suppresses fibrosis in injured hearts [58]. Another possible mechanism may be attributed to the production and release of protective cardiokines from the cardiomyocytes to enhance the survival of cardiomyocytes after injury. The evidence comes from the mice with cardiomyocyte-specific knockdown of TGFβR1, which show dramatic elevation of protective cardiokine IL-33, growth and differentiation factor 15 (GDF-15) and thrombospondin 4 (Thbs 4) after MI [59]. The elevation of these protective cardiokines reduces the apoptosis of cardiomyocytes in the infarct area and improves the survival of mice after MI. The advancing effect of PGE_2_ on cardiomyocyte replenishment may also be related to the function of PGE_2_ in promoting proliferation of adult stem cells [60, 61]. Administration of PGE_2_ to human mesenchymal stem cells maintains proliferation and self-renewal of these cells via the EP2 receptor which then enhances the production and autocrine effect of PGE_2_ itself [61]. Moreover, human cardiomyocytes stimulated with thrombin triggers the production of PGE_2_, which in turn promotes cardiomyocyte proliferation via EP2 receptors [60]. Whether PGE_2_ exerts the same proliferative effects on CPCs in the ischemic hearts requires further investigation. The function of PGE2 during inflammation and cardiac regeneration is illustrated in Fig. 2.

## Conclusions

Cardiac inflammation continues to be a viable drug target for future development of therapeutics for cardioprotection. Paradoxically, the inflammatory events that happen after MI can either induce undesired inflammatory responses that cause long term weakening of myocardium or remodel the microenvironment that is favourable for cardiac repair. Accordingly, traditional anti-inflammatory strategy is no longer feasible to achieve desired clinical outcome. Future therapeutic approaches should focus on harnessing the inflammatory events to achieve better drug efficacy, as well as modulating the inflammatory microenvironment favourable for cardiomyocyte replenishment.

## Abbreviations

BMP7: Bone morphogenetic protein 7
cAMP: cyclic AMP
CPC: Cardiac progenitor cell
CPC: Cardiac progenitor cell
CREB: cAMP responsive element binding
CRTC: CREB transcriptional coactivators
DAMP: Damage-associated molecular pattern
EP: G protein-coupled receptor E prostanoid
GDF5: Growth differentiation factor 5
IL: Interleukin
KLF4: Kruppel like factor 4
MertK: Myeloid-epithelial-reproductive tyrosine Kinase
MI: Myocardial infarction
PGE_2_: Prostaglandin E2
PKA: Protein Kinase A
PLPs: Platelet-like proteoliposomes
TAK-1: TGF β-activated kinase 1
TGF-β: Transforming growth factor-β
Thbs4: Thrombospondin 4
